# Natural killer lytic-associated molecule plays a role in controlling tumor dissemination and metastasis

**DOI:** 10.3389/fimmu.2012.00393

**Published:** 2012-12-24

**Authors:** Richard G. Hoover, Gail Gullickson, Jacki Kornbluth

**Affiliations:** ^1^Department of Pathology, Saint Louis University School of MedicineSt. Louis, MO, USA; ^2^Veterans Administration Medical CenterSt. Louis, MO, USA

**Keywords:** natural killer cells, cell-mediated cytotoxicity, ubiquitin ligase, natural killer lytic-associated molecule

## Abstract

Natural killer lytic-associated molecule (NKLAM) is an E3 ubiquitin ligase that plays a major role in the cytolytic activity of NK cells. NKLAM is rapidly synthesized and then targeted to the granule membranes of NK cells upon NK activation. Previous studies have shown an essential role for NKLAM in NK killing activity *in vitro*. These findings were extended to an *in vivo* model of NK-mediated tumor killing in which NKLAM-deficient knockout (KO) mice injected with B16 melanoma cells were found to have significantly higher numbers of pulmonary tumor nodules than wild-type (WT) mice. To further investigate the role of NKLAM and NK function in tumor immunity *in vivo*, we utilized additional tumor models to compare tumor development and progression in NKLAM KO and WT mice. Primary tumor growth, dissemination, and metastasis of RMA-S lymphoma cells and E0771 breast cancer cells were evaluated. Both tumor cell lines were stably transfected with constructs that allow expression of green fluorescent protein (GFP), which serves as a tumor-specific marker. Intravenous injection of NK-sensitive RMA-S lymphoma cells resulted in greater dissemination of lymphoma cells in NKLAM KO mice than in WT mice. Lymphoma cells were found in the lymph nodes and bone marrow (BM) of NKLAM KO mice 2 weeks after injection; few detectable tumor cells remained in WT mice. E0771 syngeneic breast cancer cells were injected into the mammary pads of NKLAM KO and WT mice. Primary tumor growth was greater in NKLAM KO than in WT mice. More significantly, there were 4–5-fold more tumor cells in the blood and lungs of NKLAM KO than in WT mice 2 weeks after injection of tumor cells into the mammary pad. These results indicate that NKLAM plays a role in tumor development *in vivo*, especially in controlling tumor dissemination and metastasis to distant sites.

## Introduction

Natural killer (NK) cells play an important role in defense against tumors, viruses, and microbial pathogens. The anti-tumor and anti-viral activities of NK cells are, in part, due to the direct cytolytic activity of these cells (Herberman and Ortaldo, 1981; Trinchieri, 1989; Lanier, 1998). NK cells are also potent producers of various cytokines, including interferon (IFN)-γ and tumor necrosis factor-α, which contribute to the role of NK cells in inflammatory and immune responses (Kasahara et al., 1983). NK cells can induce target cell death by a variety of mechanisms. However, the primary mechanism of killing is the release of cytotoxic granules containing perforin and granzymes (Russell and Ley, 2002; Stinchcombe and Griffiths, 2007). It has long been known that NK-mediated killing can be augmented by cytokines, including IL-2, IL-12, IL-15, and IFN (Ortaldo et al., 1980; Trinchieri et al., 1984; Kornbluth and Hoover, 1988; Fehniger et al., 2007). In studies to characterize additional proteins associated with the cytolytic process, we identified a novel protein whose expression was highly increased upon cytokine stimulation of NK cells (Kozlowski et al., 1999; Portis et al., 2000). This protein was named NK lytic-associated molecule (NKLAM).

NKLAM is an E3 ubiquitin ligase and a member of the RING1-in between RING (IBR)-RING2 (RBR) family of proteins (Fortier and Kornbluth, 2006). The N-terminus of NKLAM contains the three cysteine-rich domains that comprise the RBR structure and ubiquitin ligase activity (Fortier and Kornbluth, 2006). E3 ubiquitin ligases regulate the ubiquitination of proteins, catalyzing the transfer of activated ubiquitin from an E2 ubiquitin conjugating enzyme to its substrate (Pickart, 2001; Ardley and Robinson, 2005). Although ubiquitination targets cellular proteins for proteasomal degradation, it also regulates other cellular functions, including lysosomal targeting, kinase and transcription factor activation and DNA repair (Fang et al., 2003; Chen, 2005). Multiple E3 ubiquitin ligases have important roles in a variety of immune functions, including receptor-mediated signaling events and cytokine gene transcription (Rocca et al., 2001; Anandasabapathy et al., 2003; Chuang and Ulevitch, 2004; Liu, 2004; Zhao et al., 2005; Nurieva et al., 2010). We have identified NKLAM as an E3 ubiquitin ligase and are currently investigating how this enzymatic activity results in enhanced NK cytolytic function (Fortier and Kornbluth, 2006). NKLAM also has at least two predicted transmembrane domains, which is unique for E3 ubiquitin ligases (Kozlowski et al., 1999).

Consistent with its predicted protein structure, NKLAM is integrated into the cytolytic granule membranes of NK cells. It is weakly expressed in resting NK cells and unlike perforin and granzymes, there is little to no pre-formed NKLAM mRNA or protein in NK cells. Upon target cell stimulation or after treatment with cytokines that enhance NK killing, NKLAM mRNA levels in NK cells increase; protein is synthesized and becomes targeted to cytoplasmic granule membranes. As part of the Immunological Genome Project, it was found that NKLAM is transcriptionally up-regulated in Ly49H+ NK cells 1.5 days after mouse cytomegalovirus infection (Bezman et al., 2012). Studies have shown a role for NKLAM in perforin/granzyme-mediated cytolysis. Treatment of NK cells with NKLAM antisense oligonucleotides inhibits their killing activity, indicating that NKLAM participates in the cytotoxic process (Kozlowski et al., 1999; Portis et al., 2000). NK cells from NKLAM-deficient knockout (KO) mice are significantly defective in lysing tumor target cells (Hoover et al., 2009). *In vivo*, NKLAM KO mice also have reduced NK activity. This is accompanied by higher numbers of lung tumor colonies in NKLAM KO than in wild-type (WT) mice after injection with B16 melanoma cells.

Many studies have documented the anti-tumor activity of NK cells *in vivo*. NK cells have historically been found to be most effective in recognizing and killing hematopoietic tumors. However, there are studies linking higher levels of NK activity with better disease prognosis in patients with solid tumors (Ishigami et al., 2000; Villegas et al., 2002). Conversely, there is an epidemiological study showing that low NK activity in the peripheral blood is associated with increased cancer risk (Imai et al., 2000).

To further investigate the role of NKLAM in tumor immunity, we examined the role of NK cells in preventing metastasis using the NKLAM deficient mice as a model of defective NK cells. We utilized two *in vivo* tumor models to compare tumor development and progression in NKLAM KO and WT mice. The first, RMA-S, a murine T cell lymphoma, is a well-characterized NK-sensitive hematopoietic tumor (Karre et al., 1986; Kim et al., 2000; Diefenbach et al., 2001). The second, E0771, is a murine medullary breast carcinoma. This tumor spontaneously arose from a C57BL/6 mouse (Sugiura and Stock, 1952). The E0771 cell line is poorly immunogenic, estrogen receptor positive and highly prone to metastasis (Ewens et al., 2005, 2006; Gu et al., 2009). Primary tumor growth, dissemination, and metastasis of RMA-S lymphoma cells and E0771 breast cancer cells were evaluated. Results presented here indicate that NKLAM plays a role in controlling tumor development *in vivo*, especially tumor dissemination and metastasis to distant sites.

## Materials and methods

### Animals

The generation of mice deficient in NKLAM has been described previously (Hoover et al., 2009). NKLAM KO and corresponding C57BL/6 WT mice were housed in a pathogen-free facility. Mice ranged in age from 3 to 5 months old. Studies were conducted under protocols approved by the Saint Louis University and St. Louis VA Animal Care and Use Committees.

### Cell lines

RMA-S T lymphoma cells (a gift from Todd Fehniger) were cultured in RPMI 1640 media supplemented with 7.5% heat-inactivated bovine growth serum (Hyclone), 1% L-glutamine, and 1% penicillin–streptomycin. Cells were fed every other day and maintained at a concentration of approximately 2 × 10^5^ cells/mL. The C57BL/6-derived breast cancer cell line E0771 was obtained from Dr. Rong Xiang (Scripps Research Institute). Cells were split and fed twice a week in DMEM supplemented with 15% bovine growth serum.

RMA-S and E0771 cells expressing green fluorescent protein (GFP) were generated for *in vivo* studies, in which GFP serves as a tumor-specific marker. Cells were transfected with a pcDNA3 vector (Invitrogen) containing the GFP gene cloned from the pEGFP-N1 vector (Clontech). GFP-expressing RMA-S cells (RMA-S-GFP) and E0771 cells (E0771-GFP) were selected in media containing 800 μg/mL and 1 mg/mL G418, respectively. GFP expression was verified by fluorescence microscopy, flow cytometry, and PCR.

### RMA-S-GFP and intravenous (tail vein) injections

RMA-S-GFP cells (5 × 10^5^) were injected into the tail vein of WT and NKLAM KO mice. Four and twenty-four hours after intravenous injection of the cells, the mice were sacrificed and the liver, lung, and blood were harvested. In some experiments, mice were followed for 14 days after intravenous injection of tumor cells. In addition to analysis of tumor survival in the lungs, dissemination of tumor to the blood, bone marrow (BM), and lymph nodes was assessed.

### Subcutaneous injections

RMA-S-GFP cells (5 × 10^5^) were mixed 1:1 with Matrigel and injected into the left flank of WT and NKLAM KO mice. E0771-GFP cells (2 × 10^5^) were mixed 1:1 with Matrigel and injected into the mammary pads of syngeneic WT and NKLAM KO mice. Primary tumor growth was measured twice a week using calipers. Tumor volumes (mm^3^) were calculated using the formula: (width)^2^ × length/2, where width is the smaller of the two measurements. Mice were sacrificed at various days and primary tumor, blood, liver, and lungs were harvested. Primary tumors were dissected free of surrounding tissue, weighed and final tumor volumes (mm^3^) calculated using the formula: width × length × depth/2. Pieces of tumor tissue, the right apical lobe of the lung and sections of lymph node, spleen, and liver from each animal were fixed in formalin and embedded in paraffin. Sections were stained with hematoxylin and eosin (H&E) for histological examination. Results were analyzed in a blinded fashion. Lymph nodes, lung, and liver sections were also snap frozen in liquid nitrogen and stored at −70°C.

### Real time RT-PCR analyses

These studies were performed as previously described (McHowat et al., 2011). Briefly, the right azygous lobe of the lung and a section of liver were snap frozen and the tissue was homogenized using a rotor-stator homogenizer (Tissuemiser, Fisher Scientific). Blood was obtained by cardiac puncture. RNA was prepared using the RNeasy Mini Kit (Qiagen) and cDNA using the TaqMan Reverse Transcription Gene Expression Assay kit (Applied Biosystems). Real-time PCR analysis of GFP and 18 s RNA was performed using GFP and 18 s RNA-specific Taqman primer/probes and the ABI 7500 Real Time PCR System (Applied Biosystems). GFP levels were analyzed as the change in Ct (ΔCt), calculated with 18 s RNA as a housekeeping gene control. With this technique, we can detect as few as 50 tumor cells in the lungs and blood. We have shown previously that the quantitative PCR measurements correlate with H&E histological analysis; however, PCR can detect lower levels of metastasis than histology (McHowat et al., 2011).

### Flow cytometry

Blood and BM were analyzed for the presence of tumor cells by histology and flow cytometry. For isolation of BM, femurs and tibias were dissected from euthanized mice. The bones were flushed with DMEM to collect the marrow. BM cells were stained with Wright–Giemsa stain.

RMA-S and E0771 cells were evaluated for cell surface expression of MHC class I, CD45, CD62L and NKG2D ligands (NKG2DL) by flow cytometry. Briefly, tubes containing 2 × 10^5^ – 2 × 10^6^ cells in 0.05 mL of flow buffer (ice cold PBS + 1% FBS + 0.1% sodium azide) were incubated with 1 ul of Fc block (BD Pharmingen) for 10 min at 4°C. Fluorescent conjugated cell surface-specific primary antibodies were then added and incubated for an additional 30 min at 4°C. Cells were washed twice with flow buffer and analyzed on a Becton Dickinson FACScaliber flow cytometer. Antibodies PerCP CD45 and APC CD62L were purchased from BD. PerCP-eFluor 710 MHC class I antibody was purchased from eBioscience. The NKG2D/CD314 Fc chimera was purchased from R&D Systems and PE-conjugated F(ab')_2_ goat anti-human Fc fragment specific IgG was from Jackson ImmunoResearch. Flow cytometry was also performed on blood for GFP expression. For analysis of total cell counts in the blood, 10 μ L of unwashed blood was stained directly with CD45 antibody and AccuCheck counting beads (Invitrogen) were added just prior to flow analysis. Flow cytometric data were analyzed with FlowJo software (Treestar).

### Statistical analysis

A two-tailed, unpaired Student's *t*-test was used to compare the means of two groups. A *p*-value of 0.05 or lower was considered statistically significant.

## Results

### Analysis of tumor cell persistence, dissemination, and metastasis

We developed a highly sensitive and quantitative PCR assay to measure small numbers of tumor cells *in vivo* (McHowat et al., 2011). Tumor cells are stably transfected with GFP cDNA, which serves as a tumor-specific marker. Using a quantitative real time PCR assay for detection of GFP, it is possible to identify tumor cells in organs throughout the body that would otherwise be undetectable.

### Characterization of tumor cells

**Figure 1:**
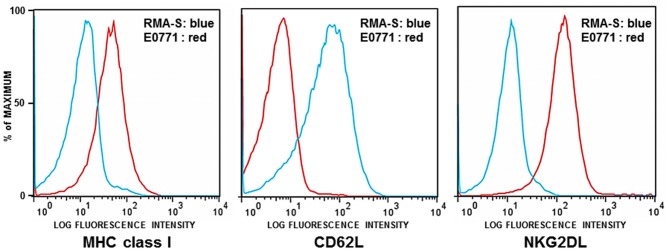
**Flow cytometric analysis of RMA-S and E0771 tumor cells.** RMA-S and E0771 cells were immunostained for cell surface expression of MHC class I, NKG2D ligands (NKG2DL), and L-selectin (CD62L). Histograms depict RMA-S in blue and E0771 in red. The X axes indicate the fluorescence intensity of staining for MHC class I, NKG2DL, and CD62L. The Y axes represent the number of cells analyzed, normalized to the percentage of maximum events.

The first set of experiments was performed using GFP-expressing RMA-S tumor cells (RMA-S-GFP). RMA-S is a variant of the Rauscher virus-induced C57BL/6 derived RBL-5 lymphoma, defective for peptide loading of MHC class I molecules. Due to its lack of stable cell surface MHC class I expression, RMA-S is an NK-sensitive target *in vitro* and *in vivo* (Karre et al., 1986; Kim et al., 2000; Diefenbach et al., 2001). As previously reported and shown here, RMA-S also lacks expression of NKG2D ligands (NKG2DL) (Cerwenka et al., 2001). It does, however, express L-selectin (CD62L), which is critical for entry of cells into lymph nodes (Figure 1).

The murine medullary breast carcinoma line E0771 spontaneously arose from a C57BL/6 mouse (Sugiura and Stock, 1952). This cell line is poorly immunogenic, estrogen receptor positive and highly metastatic (Ewens et al., 2005, 2006; Gu et al., 2009). As shown in Figure 1, E0771 expresses MHC class I and NKG2D ligands but lacks cell surface expression of CD62L.

### NKLAM plays a role in lymphoma survival and dissemination

**Figure 2:**
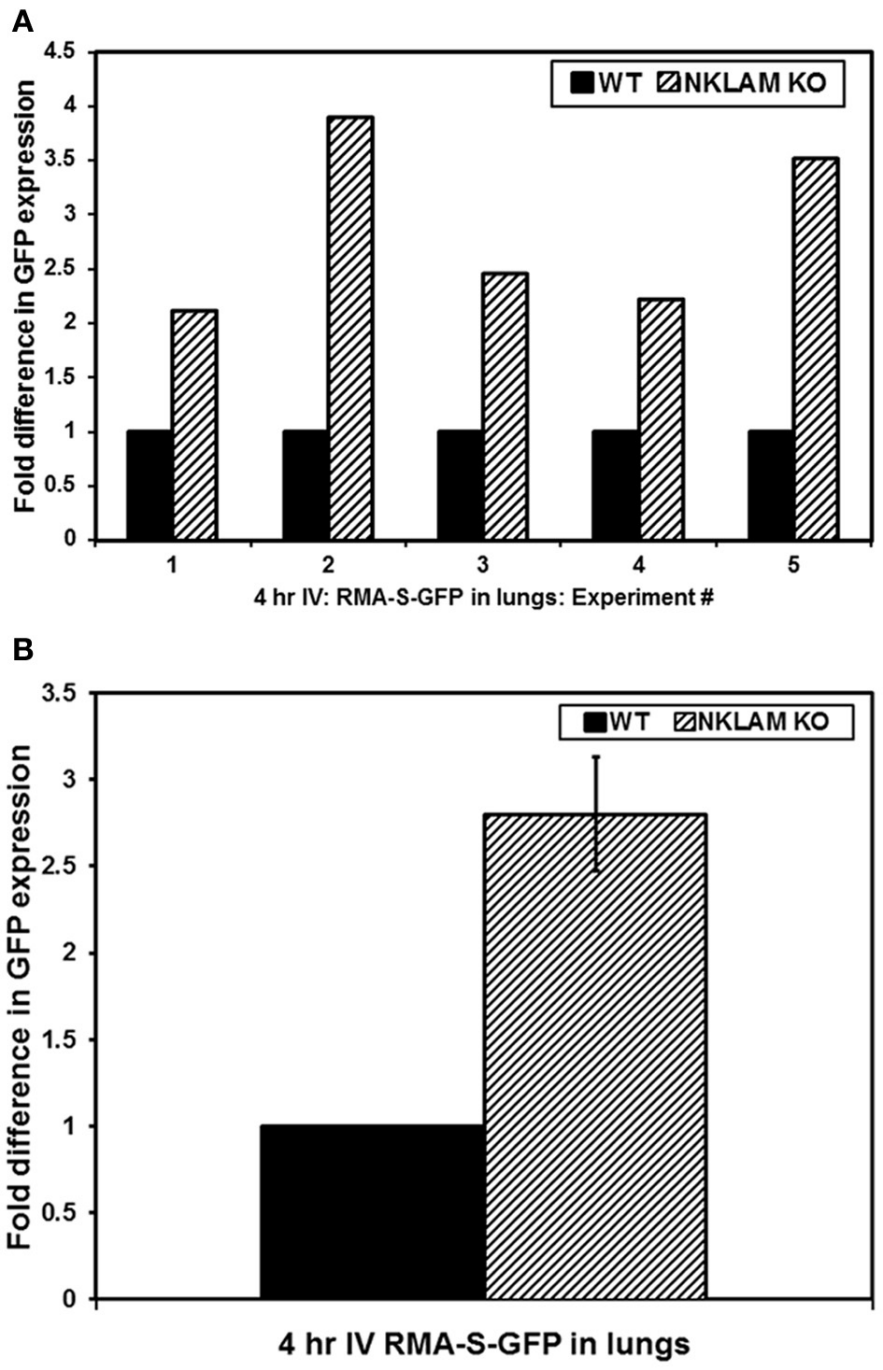
**Greater persistence of RMA-S-GFP cells in the lungs of NKLAM KO than WT mice.** Four hours after intravenous injection of RMA-S-GFP cells, lungs from NKLAM KO and WT mice were analyzed for GFP expression by quantitative PCR. **(A)** Depicts the results of five independent experiments. Experiment 1: WT mice *n* = 3, NKLAM KO mice *n* = 3; Experiment 2: WT *n* = 6, NKLAM KO *n* = 6; Experiment 3: WT *n* = 6, NKLAM KO *n* = 4; Experiment 4: WT *n* = 4, NKLAM KO *n* = 4; Experiment 5: WT *n* = 5, NKLAM KO *n* = 6. **(B)** Depicts the compilation of the five experiments (WT mice *n* = 24, NKLAM KO mice *n* = 23) (*p* < 0.02).

NKLAM KO and WT mice were injected with RMAS-GFP lymphoma cells intravenously. Four hours later, tumor survival in the lungs was assessed by quantitative PCR, in which GFP PCR levels in the lungs represent the number of tumor cells surviving. Results of five independent experiments as well as the compilation of the five experiments are shown in Figures 2A,B, respectively. We found that 2.8 times more RMAS-GFP tumor cells survived in the lungs of NKLAM KO mice compared to WT mice 4 h after intravenous injection (*p* < 0.02). This represents an 11-fold decrease in tumor burden in NKLAM KO mice compared to a 30-fold decrease in WT mice.

**Figure 3:**
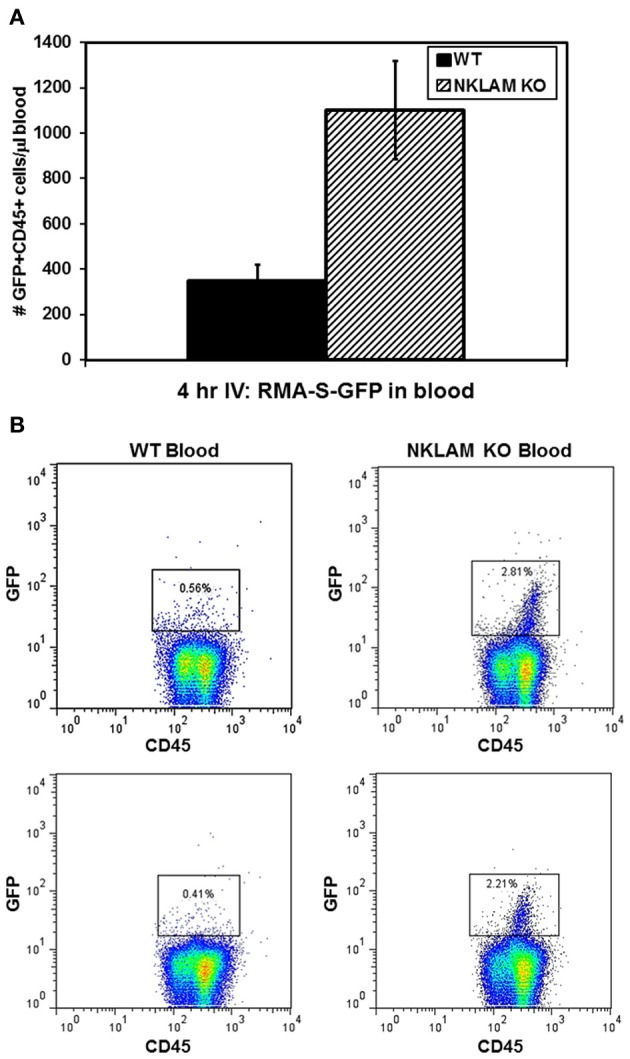
**Higher levels of circulating RMA-S-GFP cells in the blood of NKLAM KO than WT mice.** Four hours after intravenous injection of RMA-S-GFP cells, blood from NKLAM KO (*n* = 5) and WT mice (*n* = 5) were analyzed for GFP expression by flow cytometry. **(A)** Results are expressed as the number of GFP+ CD45+ cells per μL of blood. (*p* < 0.02). **(B)** Representative histogram plots of blood from NKLAM KO and WT blood are shown. Tumor cells (GFP+ CD45+) are within the box. The percentage of GFP+ RMA-S cells within the CD45+ population in each sample is shown.

We also examined the blood of these mice 4 h after injection of tumor cells for the presence of circulating GFP+ RMA-S cells by flow cytometry. CD45 was used to gate the total leukocyte population in the blood, inclusive of RMA-S. There were very few, but detectable, tumor cells in the circulation. WT mice had an average of 349 GFP+ cells within the CD45+ population per μL of blood while NKLAM KO mice had an average of 1100 GFP+ cells, representing over 3-fold (3.2) more tumor cells than WT (*p* < 0.02) (Figure 3A). Representative flow analyses are shown in Figure 3B. By quantitative real-time PCR analysis of GFP levels in the blood, a similar (2.9-fold) difference in tumor levels between NKLAM KO and WT mice was observed (*p* < 0.04). These results indicate that NKLAM KO mice are less capable than WT mice of eliminating RMA-S tumor cells from the lungs and the circulation.

**Figure 4:**
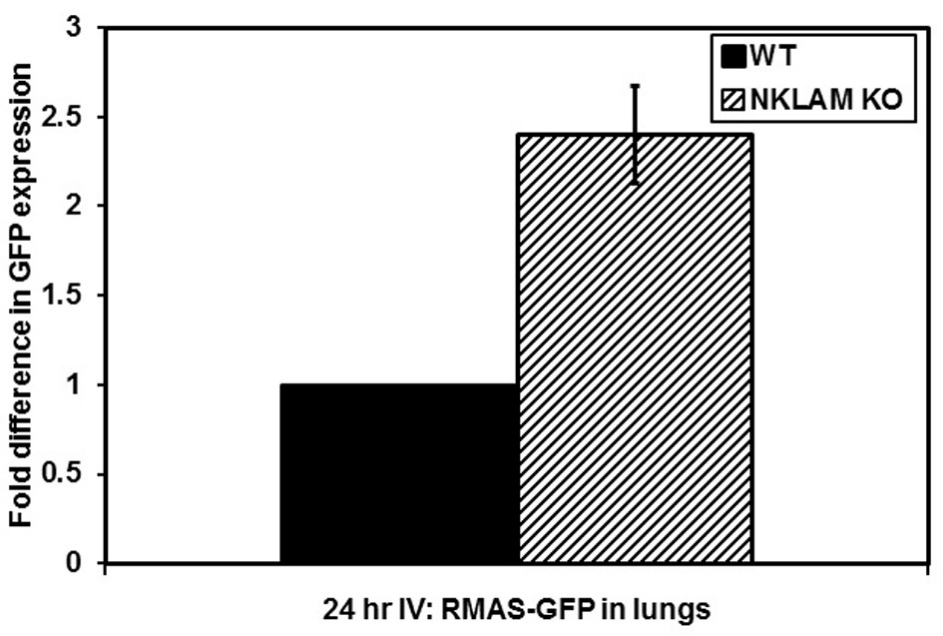
**Persistence of RMA-S-GFP cells in the lungs of NKLAM KO mice 24 h after intravenous injection.** Lungs from tumor-injected NKLAM KO (*n* = 4) and WT mice (*n* = 4) were analyzed for GFP expression by quantitative PCR (*p* < 0.05).

The elimination of RMA-S cells *in vivo* is extremely rapid. Over 90% of the cells that reach the lung after intravenous (tail vein) injection are killed within 4 h in WT mice. To evaluate the persistence of tumor cells beyond this early time point, we utilized PCR to identify RMA-S-GFP cells in the lungs and blood of mice 24 h after intravenous injection. We were able to detect a low GFP signal in the lungs of both NKLAM KO and WT mice by PCR. Again, NKLAM KO mice had 2.5-fold more RMA-S cells persisting in the lungs than WT mice 24 h after injection (*p* < 0.05) (Figure 4).

**Figure 5:**
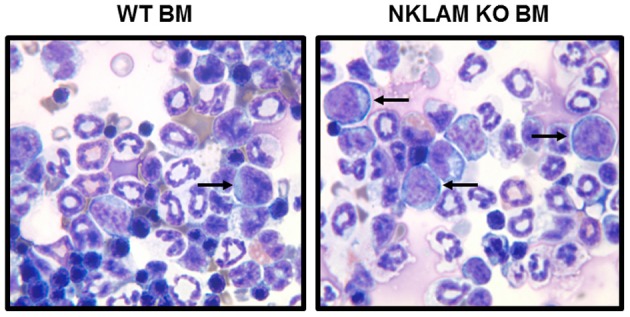
**Greater numbers of RMA-S-GFP cells disseminate to the bone marrow (BM) of NKLAM KO than WT mice 14 days after intravenous injection.** BM from NKLAM KO (*n* = 6) and WT mice (*n* = 8) was harvested 14 days after tumor injection and stained with Wright Giemsa stain. Arrows indicate the RMA-S tumor cells. Based upon standard differential counts, there were 26-fold more tumor cells present in the BM of NKLAM KO mice than WT mice (*p* < 0.001).

Since we were able to detect a small number of RMA-S tumor cells persisting in the lungs of mice 24 h after intravenous injection, we next evaluated whether any of these cells ultimately disseminated to lymphoid or other organs. Accordingly, we examined a series of NKLAM KO and WT mice 14 days after intravenous injection of RMA-S-GFP cells. At 14 days, we were unable to detect any RMA-S lymphoma cells in the lungs by PCR. Lung weights were equivalent between NKLAM KO and WT mice. The lymph nodes were examined for evidence of lymphoma dissemination. Of great significance was the finding that 88% of NKLAM KO mice had tumor cells in their lymph nodes, while less than 25% of WT mice were lymph node positive. BM from NKLAM KO and WT mice was also examined for the presence of tumor cells. BM smears were made and scored blind by a hematopathologist. Differential counts were performed to quantitate the number of tumor cells in each WT and KO BM sample. BM from six WT and eight NKLAM KO mice were evaluated. The average percentage of RMA-S lymphoma cells in the BM of WT mice was 0.8% (range 0.17–1.5%); the average in NKLAM KO mice was 20.9% (range 16.5–26%). This represents a 26-fold difference in tumor burden (*p* < 0.001). Two examples are shown in Figure 5.

**Figure 6:**
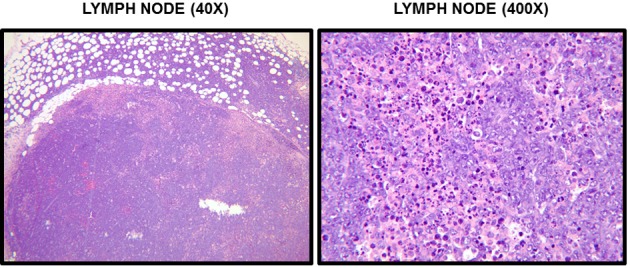
**Histological examination of RMA-S tumors.** Sections of lymph nodes from mice 20 days after subcutaneous injection of RMA-S were stained with H&E. A section of tumor-involved lymph node from an NKLAM KO mouse at 40× and 400× magnification is shown. At 40×, it is evident that the normal lymph node architecture is completely effaced and tumor cells have extended through the capsule into the perinodal adipose tissue. Histological examination at 400× reveals extensive replacement of the node by large, anaplastic RMA-S tumors cells and many apoptotic cells. These findings are indicative of replacement of the lymph node by a rapidly proliferating lymphoma.

In the next series of experiments, RMA-S-GFP cells were injected subcutaneously into NKLAM KO and WT mice in order to compare primary tumor growth rates. At sacrifice on day 20, we found no significant difference in primary tumor weight between NKLAM KO and WT mice. However, 80% of NKLAM KO mice had evidence of tumor in their lymph nodes while only 27% of WT mice were lymph node positive (*p* = 0.0001) (Table 1). Positive lymph nodes recovered from WT and KO mice on day 20 were completely replaced by RMA-S lymphoma cells. Representative histology of a tumor-positive lymph node from a KO mouse is shown in Figure 6. Nests and sheets of lymphoma cells are seen, the lymph node architecture is effaced, and lymphoma cells have extended through the lymph node capsule into the perinodal adipose tissue. Lymph nodes recovered from WT mice showed similar histology. Cumulatively, these results suggest that NKLAM KO mice are less capable of controlling lymphoma dissemination than WT mice.

**Table 1:** **Primary tumor weight and lymph node dissemination of RMA-S tumors 21 days after subcutaneous injection**.

	**Tumor weight (g)**	**Frequency of tumor in lymph nodes**
WT	2.6 ± 0.21 (*n* = 18)	3/11
NKLAM KO	2.4 ± 0.21 (*n* = 21)	12/15^*^

Data represent means ± SE;

* p < 0.0001.

### NKLAM plays a role in control of breast cancer metastasis

**Figure 7:**
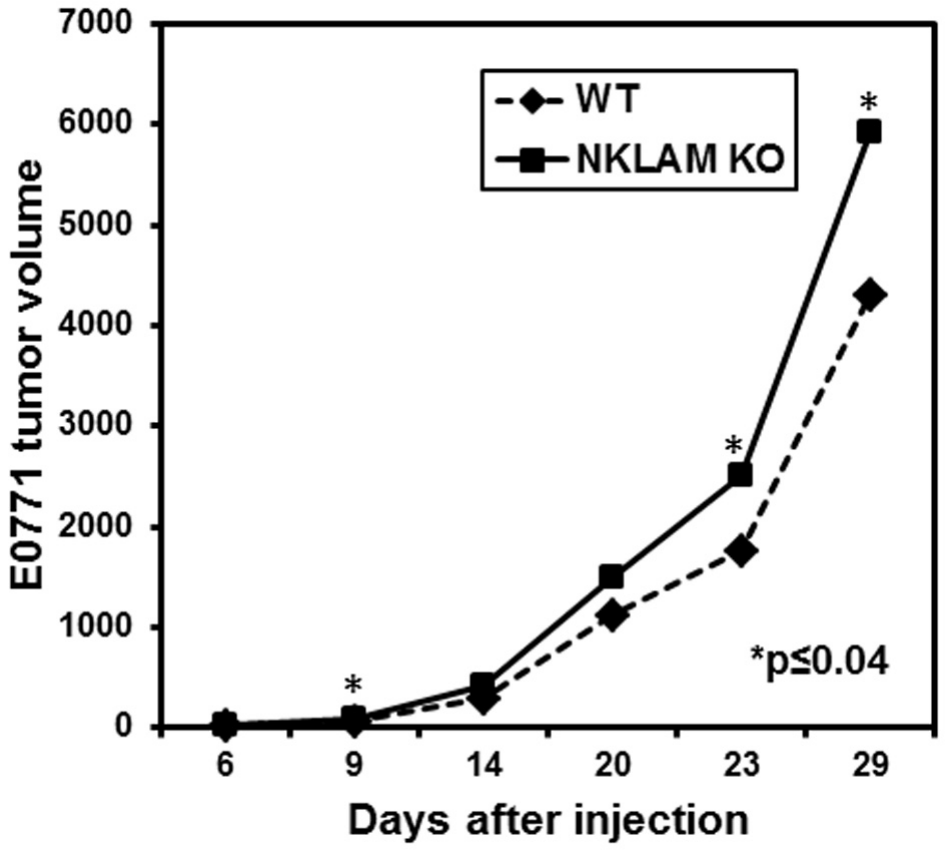
**Growth of E0771 primary tumors in NKLAM KO and WT mice.** GFP-expressing E0771 cells were injected into the mammary pads of female mice. Mice were observed for up to 29 days and primary tumors were measured at regular intervals with calipers (NKLAM KO mice, *n* = 21; WT mice, *n* = 18). Tumor volume is expressed as mm^3^ (^*^p ≤ 0.04).

To examine the potential role of NKLAM in controlling breast cancer growth and metastasis, mammary pads of WT and NKLAM KO female mice were injected with 200,000 GFP-expressing E0771 cells. Mice were observed for up to 29 days and primary tumor volume (mm^3^) measured at regular intervals (Figure 7). Tumor growth was slightly higher in NKLAM KO than in WT mice. The tumor weights at sacrifice were also somewhat higher in NKLAM KO mice, but the differences were not statistically significant.

**Figure 8:**
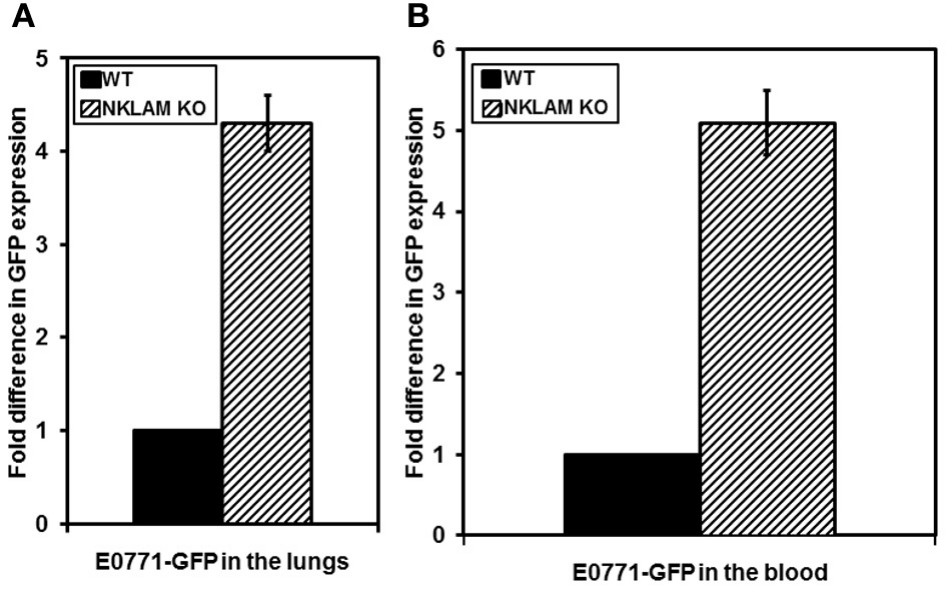
**Higher levels of metastasis of E0771 in NKLAM KO than WT mice. (A)** Lungs and **(B)** blood were collected from mice 24 days after primary breast tumor growth. Tumor metastasis and dissemination were evaluated by quantitative PCR for GFP expression. WT mice, *n* = 5; NKLAM KO mice, *n* = 6 (**A**, *p* < 0.05; **B**, *p* = 0.02).

**Figure 9:**
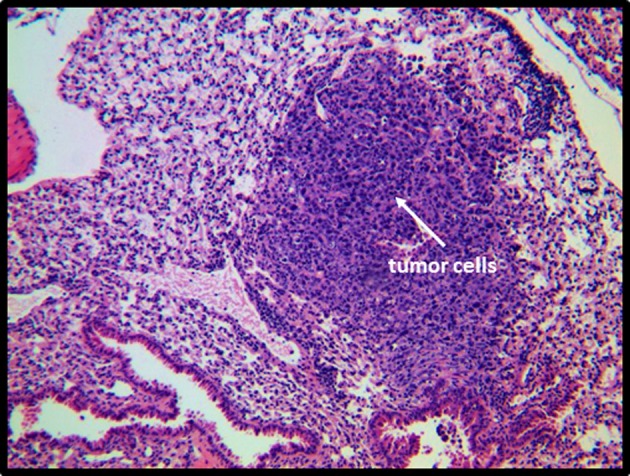
**Histological analysis of a lung from an NKLAM KO mouse 24 days after mammary pad injection of E0771.** Lungs were collected from mice 24 days after primary tumor growth and sections stained with H&E. A focus of E0771 cells in the lung of a mouse with a high level of metastasis is depicted. The surrounding, uninvolved lung tissue shows atelectasis and infiltration by acute and chronic inflammatory cells (primarily mature granulocytes and small lymphocytes and monocytes).

E0771 breast cancer cells often metastasize to distant sites. To compare levels of metastasis in NKLAM KO and WT mice, a series of mice were sacrificed 24 days after tumor initiation. Primary tumor, blood, liver, and lungs were harvested. Lungs, liver, and blood were analyzed for tumor metastasis by quantitative RT-PCR analysis of GFP, which can identify metastasis that would otherwise be undetectable by other methodologies. We were unable to detect tumor cells in the liver of these mice. However, there was significant GFP expression, reflecting tumor cells, in the blood and lungs of all mice. NKLAM KO mice had over four times more E0771 cells in the lungs and over five times more E0771 cells in the blood than WT mice (Figure 8). These differences were highly significant (*p* ≤ 0.02). Figure 9 represents histological analysis of a lung from an NKLAM KO mouse with a high level of metastasis, as determined by PCR analysis of GFP levels. A focus of E0771 tumor cells is clearly identifiable in this section of lung. However, overall, the level of tumor metastasis to the lung in mice at this time point was too low to detect histologically in the majority of animals. Real-time quantitative PCR analysis of GFP levels in the blood and lung allowed for the detection of micro-metastases which were undetectable by histology. Therefore, although primary breast cancer tumor growth is only slightly higher in NKLAM KO mice compared to WT, NKLAM KO mice have significantly higher levels of lung metastasis and more tumor cells disseminated through the bloodstream than WT mice.

## Discussion

Studies were performed to further investigate the role of NKLAM in tumor immunity. NKLAM-deficient mice have defective NK function and therefore act as a model to evaluate the role of NK cells in tumor metastasis and dissemination. Two *in vivo* tumor models were utilized to compare both tumor development and its progression in NKLAM KO and WT mice. One is the RMA-S T cell lymphoma model. RMA-S is a well-described NK-sensitive target *in vitro* and *in vivo*. Intravenous injection of NK-sensitive RMA-S lymphoma cells through the tail vein resulted in greater persistence of tumor cells in the lungs of NKLAM KO mice compared to WT mice. NKLAM KO mice also had higher levels of tumor cells in the circulation. Over time, lymphoma cells disseminated to the lymph nodes and BM of NKLAM KO mice while few to no tumor cells were detectable in WT mice. These results suggest that NKLAM KO mice are less capable of killing RMA-S tumor cells *in vivo*, thereby allowing surviving tumor cells to disseminate to distant sites.

The potential role of NKLAM in the process of lymphoma dissemination was further examined by injecting RMA-S-GFP cells into mice subcutaneously. Studies have shown that low numbers of RMA-S cells are rejected by immunocompetent mice. At high doses (10^5^ and above), tumors develop in nearly 100% of mice (Screpanti et al., 2001). In the studies presented here, mice were injected with a dose of tumor cells that generates tumors in all mice. Primary tumor growth rate and tumor weights were equivalent in NKLAM KO and WT mice. However, dissemination of lymphoma to the lymph nodes was observed in the majority (80%) of NKLAM KO mice, while only 27% of WT mice were lymph node positive. These results additionally support a role for NKLAM in lymphoma dissemination *in vivo*.

Breast cancer growth and metastasis was the second tumor model used to evaluate the potential role of NKLAM in tumor immunity. Breast cancer is the second leading cause of death from cancer in women. Metastatic breast cancer, at time of diagnosis, is a negative prognostic indicator, and distant site metastasis is a major cause of death in cancer patients. For these studies, we injected female mice with syngeneic E0771 breast cancer cells. E0771 tumors are estrogen receptor positive, poorly immunogenic, aggressive, and metastatic, with characteristics that closely mirror those of the human disease. They are also CD73 positive, which is associated with a pro-metastatic phenotype in breast cancer and escape from anti-tumor immunity (Stagg et al., 2010). Mammary pad injection of E0771 tumor cells results in tumor formation in almost 100% of mice. By histological analysis, it has been reported that secondary tumors form in the lungs of 52% of mice (Ewens et al., 2005). However, this is likely a conservative estimate. By PCR analysis, we detect some level of metastasis to the lung in nearly all mice. Human breast cancer often metastasizes to the lungs, suggesting that E0771 may be a better model of human disease than other subcutaneously grown tumors.

Primary tumor growth and metastasis of E0771 breast cancer cells was evaluated. Primary tumor growth was slightly greater in NKLAM KO than in WT mice. The dose of E0771 cells injected into the mammary pads of mice in these experiments is high, resulting in tumors in 100% of mice. It is possible that differences in primary tumor growth between NKLAM KO and WT mice would be more apparent by injecting fewer tumor cells. To address this, limiting dilution analyses are now in progress. Of great interest is the finding of significant differences in tumor metastasis between NKLAM KO and WT mice. There were 4–5-fold more tumor cells in the blood and lungs of NKLAM KO than in WT mice 2 weeks after injection of tumor cells into the mammary pad.

The pathogenesis of metastasis is complex, requiring a series of events, including synthesis and secretion of angiogenic factors to promote neovascularization, motility, local invasion of the stroma, extravasation and entry into the circulation. Our working hypothesis is that NKLAM, through its role in NK-mediated cytotoxicity, influences the ability of tumor cells to survive in the circulation, thereby limiting tumor dissemination and metastasis. This would establish NK anti-tumor activity as a mechanism for controlling the process of tumor spread. Others have presented data consistent with a role for NK cells in controlling metastasis. Using a murine breast cancer model, Tkach et al. found that immunization of Balb/c mice with tumor cells expressing dominant-negative Stat3 decreased the level of tumor metastasis. They demonstrated that depletion of NK cells abrogated the anti-tumor effect of immunization and that vaccination increased the number of tumor-infiltrating NK cells (Tkach et al., 2012). The metastasis-associated microRNA miR-10b was found to downregulate MICB, thereby impairing the ability of tumor cells to be eliminated by NK killing. Conversely, using an *in vivo* model of lung metastasis, more tumor cells overexpressing miR-10b were present in the lungs compared with control tumor cells (Tsukerman et al., 2012). These studies demonstrate a link between NK cytotoxicity and control of metastasis. Our results are consistent with this hypothesis for the following reasons. In the first tumor model, RMA-S is a well-characterized NK sensitive target. Due to its lack of cell surface MHC class I expression, little to no cytotoxic T cell (CTL) activity is generated against these cells (Cerwenka et al., 2001; Zompi et al., 2003). The differences between NKLAM KO and WT mice in control of RMA-S *in vivo* are seen within 24 h, long before adaptive immunity would be generated. The finding that dissemination of these lymphoma cells to the BM and lymph nodes is seen predominantly in NKLAM KO mice is consistent with the hypothesis that NKLAM KO NK cells are less capable of killing RMA-S tumor cells *in vivo*, thereby allowing surviving tumor cells to disseminate to distant sites. In the second tumor model, E0771 breast cancer cells are poorly immunogenic and incapable of generating primary CTL responses *in vivo* (Ewens et al., 2005, 2006; Zhou et al., 2005). Their expression of CD73 contributes to their inability to generate adaptive anti-tumor immunity (Stagg et al., 2010). However, we have shown that these cells express high levels of NKG2D ligands, which allows them to be potential NK targets. Our results suggest that NK cells may be relatively ineffective in controlling primary breast tumor growth in the mammary pad, particularly given the large number of tumor cells that were injected in these studies. However, NK cells may be able to identify and kill the small numbers of tumor cells that escape from the mammary pad into the circulation, thereby reducing the incidence of metastasis. NKLAM KO NK cells are also defective in IFN-γ production, so it is possible that IFN may also contribute to these effects.

Cancer is the second most prevalent cause of death, and the majority of cancer deaths are caused by distant metastases rather than by growth of the primary tumor (Heyder et al., 2005). Development of new management strategies that target metastasis development will improve cancer morbidity and mortality outcomes. Further delineation of the precise mechanism by which NKLAM functions to prevent tumor dissemination and metastasis is currently under investigation.

### Conflict of interest statement

The authors declare that the research was conducted in the absence of any commercial or financial relationships that could be construed as a potential conflict of interest.
